# Genesis of Non-Coding RNA Genes in Human Chromosome 22—A Sequence Connection with Protein Genes Separated by Evolutionary Time

**DOI:** 10.3390/ncrna6030036

**Published:** 2020-09-03

**Authors:** Nicholas Delihas

**Affiliations:** Department of Microbiology and Immunology, Renaissance School of Medicine, Stony Brook University, Stony Brook, New York, NY 11794-5222, USA; Nicholas.delihas@stonybrook.edu

**Keywords:** de novo gene birth, gene evolution, protogene, long noncoding RNA genes, pseudogenes, *USP18*, *GGT5*, *Alu* sequences

## Abstract

A small phylogenetically conserved sequence of 11,231 bp, termed FAM247, is repeated in human chromosome 22 by segmental duplications. This sequence forms part of diverse genes that span evolutionary time, the protein genes being the earliest as they are present in zebrafish and/or mice genomes, and the long noncoding RNA genes and pseudogenes the most recent as they appear to be present only in the human genome. We propose that the conserved sequence provides a nucleation site for new gene development at evolutionarily conserved chromosomal loci where the FAM247 sequences reside. The FAM247 sequence also carries information in its open reading frames that provides protein exon amino acid sequences; one exon plays an integral role in immune system regulation, specifically, the function of ubiquitin-specific protease (USP18) in the regulation of interferon. An analysis of this multifaceted sequence and the genesis of genes that contain it is presented.

## 1. Introduction

The genesis of genes has been a major topic of interest for several decades [1,2]. One mechanism of gene formation is by duplication of existing genes [1,3]. This is considered one of the major processes in protein gene development, but it has also been shown that there is a prevalence of gene birth from noncoding DNA via de novo processes [4,5,6,7]; this pathway also significantly contributes to new protein gene formation [4,7]. Working with yeast *Saccharomyces cerevisiae* genomic segments, Carvunis et al. [4] formulated an evolutionary model for the de novo development of protein genes in genetic regions where there are no annotated genes but where there is the translation of small open reading frames. These regions are considered protogene elements that can develop into functional genes. With respect to long noncoding RNA (lncRNA) genes, Ulitsky and Bartel [8] have provided a comprehensive background on lncRNA transcripts and genes that includes a discussion of mechanisms of lncRNA gene origins where some gene birth processes may be similar to those that operate in protein gene formations.

In this treatise, we analyze the development of long intergenic noncoding RNA (lincRNA) genes and pseudogenes by an evolutionarily conserved ancestral sequence. This is a repeat element found in human chromosome 22. It was previously termed clincRNA [9] and is now officially termed FAM247 by the HUGO Gene Nomenclature Committee (https://www.genenames.org/), and it constitutes the *FAM247A-D* gene family. The *FAM247A* gene sequence has previously been used as a guide to finding homologous sequences [9]. Heretofore, FAM247 is used in place of *FAM247A*.

We previously proposed that FAM247 carries information to form a nucleation site for gene development [9]. This is best exemplified with the formation of pseudogenes by the addition of extraneous chromosomal sequences to specific sites on FAM247, a process that is now described here in this current paper. This is a model that perhaps can be considered analogous to the model of de novo protein gene development via a protogene element [4], with the FAM247 sequence serving as the protogene. During the formation of these pseudogenes, sequences that consist of full or partial copies of unprocessed parent protein genes are added to the FAM247 protogene, as well as sequences that are added from other unrelated genomic regions to form the final gene sequence.

Both the pseudogenes and the *FAM247A-D* lincRNA family genes appear to be human-specific. The FAM247 sequence is also found in protein genes *USP18* (ubiquitin-specific protease) and *GGT5* (gamma glytamyltransferase). Both these genes date back in evolutionary time, *USP18* over 350 million years ago (MYA) and *GGT5* over 90 MYA. Thus, the FAM247 sequence has formed a part of genes through much of vertebrate evolution, and it continues to do so. The FAM247 sequence is of particular interest because of its evolutionary conservation and its presence in diverse genes, from humans to zebrafish. In addition, the FAM247 sequence carries information in terms of its open reading frames where the open reading frames are found to form exon sequences of proteins, the most important being the carboxy terminal amino acid sequence of USP18.

## 2. Background on Conserved Linked Sequences

**Figure 1:**
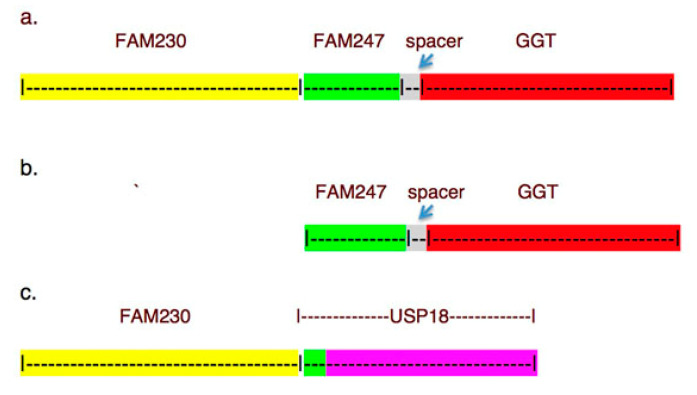
Schematic representation of evolutionarily conserved linked sequences with different colors depicting different sequences and family genes, as described in [9]. (**a**,**b**) Conserved sequences that are found linked to GGT sequences. (**c**) The FAM247 sequence (green highlight) is embedded in the *USP18* gene.

FAM247 is present in different segmental duplications or low copy repeats (LCR22) in human chromosome 22 (chr22) as part of phylogenetically conserved linked gene sequences [9]. Figure 1 is a representation of these linked sequences, and it shows conserved nearest neighbor sequence signatures found in humans. The linked gene sequences are repeated in chr22 and generate gene families. The signatures are also representative of ancestral primate linked sequences, e.g., the sequence arrangement in Figure 1b is present in the Rhesus Old World monkey (*Macaca mulatta*), where FAM247 and spacer sequences are linked to *GGT1* on chr10. The spacer sequence (3953 bp) depicted in Figure 1 is also conserved in primates. It is present in *Pan troglodytes* (chimpanzee), *Papio Anubis* (olive baboon), *Pongo abelii* (Sumatran orangutan), and *Macaca mulatta* (Rhesus monkey) genomes, but it does not encode genes or form part of genes. This conservation indicates it may have a function. FAM247 is the common denominator in Figure 1a,b. In Figure 1c, the FAM247 sequence depicted is embedded in the *USP18* gene.

Table 1 contains a list of human gene families that are found in repeat units shown in Figure 1 and indicates the sequence or chromosomal locus of origin. For example, GGT represents the locus of origin of *GGT1*, the gamma-glutamyltransferase and gamma-glutamyltransferase light chain genes and their respective pseudogenes; FAM247 is the sequence/locus of origin of *GGT5*.

A description of genes is as follows. *POM121L9P* and *POM121L10P* are members of the POM121 transmembrane nucleoporin like 1 pseudogene family. *BCRP3* is a member of the BCR pseudogene family. *BCR*, a large gene of 137,529 bp, is the activator of RhoGEF and GTPase and was formerly termed breakpoint cluster region protein. The *BCR* gene is important clinically as it is associated with the production of the Philadelphia chromosome in chronic myelogenous leukemia [10,11]. *POM121L9P* and *BCRP3* stem from the FAM247 sequence at chromosomal loci, where the GGT sequence is found, as represented in Figure 1b. *USP18* is the ubiquitin-specific peptidase gene, a member of the deubiquitinating protease family; the protein product plays a major role in interferon regulation [12], and it has multiple functions [13].

## 3. lincRNA Gene Families

The *FAM230* lincRNA and *FAM247* lincRNA gene families were named by the HUGO Gene Nomenclature Committee (https://www.genenames.org/) [14]. These genes exemplify how segmental duplications or low copy repeats in chromosome 22 are a driving element in the genesis and proliferation of lincRNA gene families. Ten *FAM230* and five *FAM247* genes are present in chr22 low copy repeats (LCR22) that originate from sequence duplications [9]. *FAM230* family genes differ from one another in sequence, transcript sequence and exon number, and RNA expression in various fetal developing tissues [9,15,16]. Their functions are not known. FAM230 sequences are also present in primates, but these sequences are annotated as predicted protein genes or pseudogenes, not as lincRNA genes, e.g., more than eleven genes that contain the FAM230 sequence in chimpanzee and gorilla are annotated as protein genes and two FAM230 sequences in Rhesus monkey and olive baboon are annotated as pseudogenes. An example is *LOC106992440*, which is found in the Rhesus monkey, annotated as an uncharacterized pseudogene, and resides in a chr locus that is homologous to that of human *FAM230D* linked to *USP18* in humans; the Rhesus *LOC106992440* has 58% identity with the human lincRNA *FAM230D*. In this and other cases, the detection of an experimental transcript that verifies the computational prediction of a protein gene or pseudogene is essential. Evolutionarily, the FAM230 sequence may have originated in the Rhesus monkey or Old World monkeys as the FAM230 sequence is present in the Rhesus genome but is not found in the genome of the Prosimian primate ancestor, *Philippine tarsier*.

The *FAM247* lincRNA gene family may have newly formed in humans as there are few or no differences in gene sequence or RNA transcript expression in somatic and fetal developing tissues [9,15]. A homologous sequence to FAM247 is present in chimpanzee and is linked to *GGT2*. It has the full-length FAM247 sequence [9], but the chimpanzee sequence has not been annotated as a gene. Segments of sequences homologous to FAM247 are found in other primate genomes (gorilla, orangutan, Rhesus monkey, *Philippine tarsier*), and the FAM247 sequence may date back evolutionarily to the house mouse and zebrafish (discussed below).

## 4. The FAM247 Sequence is Present in Diverse Genes

**Figure 2:**
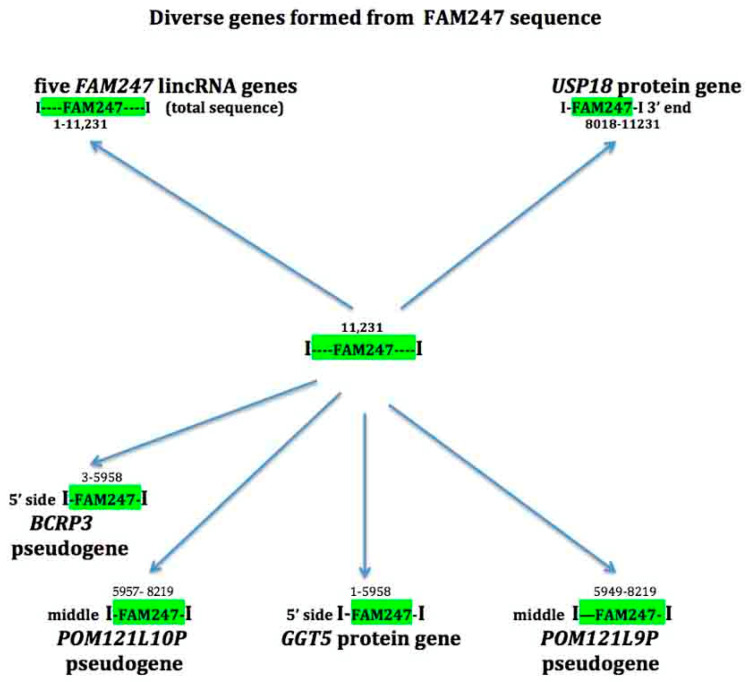
Protein genes, lincRNA genes, and pseudogenes that stem from the FAM247 sequence and contain different sections of the FAM247 sequence (shown in bp position numbers above FAM247 highlighted in green). Analysis of FAM247 by the RepeatMasker program: RepeatMasker http://www.repeatmasker.org/cgi-bin/WEBRepeatMasker shows the presence of *Alu* sequences in the FAM247 sequence at bp positions 6007–6285 and 8063–8374, the regions close to breaks. The segment that includes bp positions 8063–8374 of FAM247 has seven *Alu* elements in tandem repeats.

A significant property of the FAM247 sequence is that it forms part of diverse genes. Sequences homologous to FAM247 form genes that include lincRNA genes, pseudogenes, and protein genes (Figure 2). These genes stem from phylogenetically conserved nearest neighbor gene loci, where the FAM247 sequence is linked to adjacent genes that form signatures containing gene families, e.g., *FAM230E*-*FAM247C*-*GGT3P* [9] present in segmental duplication LCR22A and *FAM230B-FAM247A-GGT2* in LCR22D. Other than the FAM247 lincRNA family genes, which contain the entire 11,231 bp, only segments of FAM247 are found to be part of other genes. The ends of these segments may represent sequence breaks, i.e., bp positions ~5958 and ~8000–8200 (Figure 2, see numbers above green highlighted FAM247). These are regions that contain *Alu* sequences (Figure 2, caption), and may provide sites for attachment of other sequences to FAM247. FAM247 contains a total of fifteen *Alu* elements.

### 4.1. USP18

**Figure 3:**
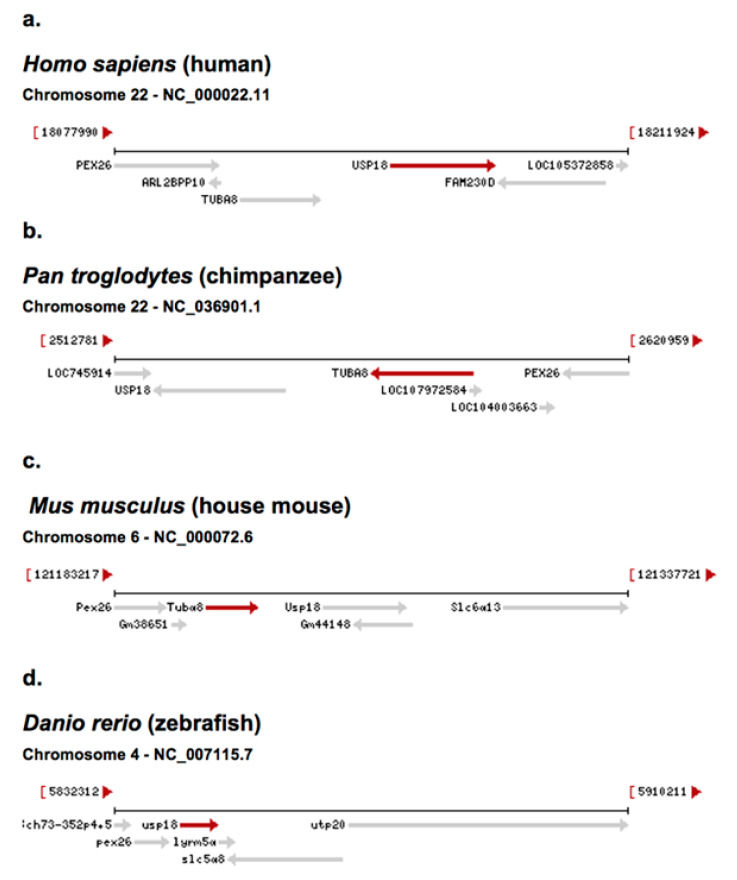
Genes that are adjacent to *USP18* are found in different species and are shown in the chromosomal and gene maps. (**a**–**d**) Drawings of gene arrangements are taken directly from the NCBI website: https://www.ncbi.nlm.nih.gov/gene [17].

A comparison of *USP18* chromosomal coordinates at loci in different species shows that the position is evolutionarily conserved relative to adjacent genes (Figure 3). This provides nearest neighbor signatures where evolutionary history (genetic synteny) and origins of *USP18* can be assessed. Two neighbor genes, *PEX26* (peroxisomal biogenesis factor 26) and *TUBA8* (tubulin alpha 8) are in homologous loci that show a conserved orientation with respect to each other in the chromosomes of mice (*Mus musculus* (house mouse)) and primates (Figure 3a–c). In zebrafish (*Danio rerio*, a member the Cyprinidae family of freshwater fish), the tubulin gene (termed tuba8l4 tubulin, alpha 8 like 4) appears to have moved to a different chromosome and developed into two genes, *TUBAa* and *TUBAb*; this results in *PEX28* and *USP18* as immediate neighbor genes in zebrafish chr4 (Figure 3d). The nearest neighbor history of the *USP18* gene locus and the display of evolutionary conservation of gene positions relative to each other are consistent with a common lineage of the *USP18* gene. Previously, Ulitsky and Bartel [8] provided an interesting analysis of human, mouse, and zebrafish vertebrate genomes with respect to the concept of synteny of genetic loci and the lineage of lincRNA genes through evolution.

To analyze the phylogenetic relatedness of *USP18* gene nt and aa sequences, sequences were aligned from zebrafish, the house mouse, three primate species, and humans. The resultant percent sequence identities mimic evolutionary distances between the species (Table 2), with a linear change in nt and aa sequences with time between the primate and mouse species (not shown). The pattern shows a continuum of gene nt and protein aa sequence change with evolutionary time and is consistent with a common lineage of the *USP18* gene that dates to an ancestor of zebrafish, more than 350 million years ago (MYA). This parallels the nearest neighbor gene history of *USP18.*

### 4.2. USP18 Exon 11

**Figure 4:**
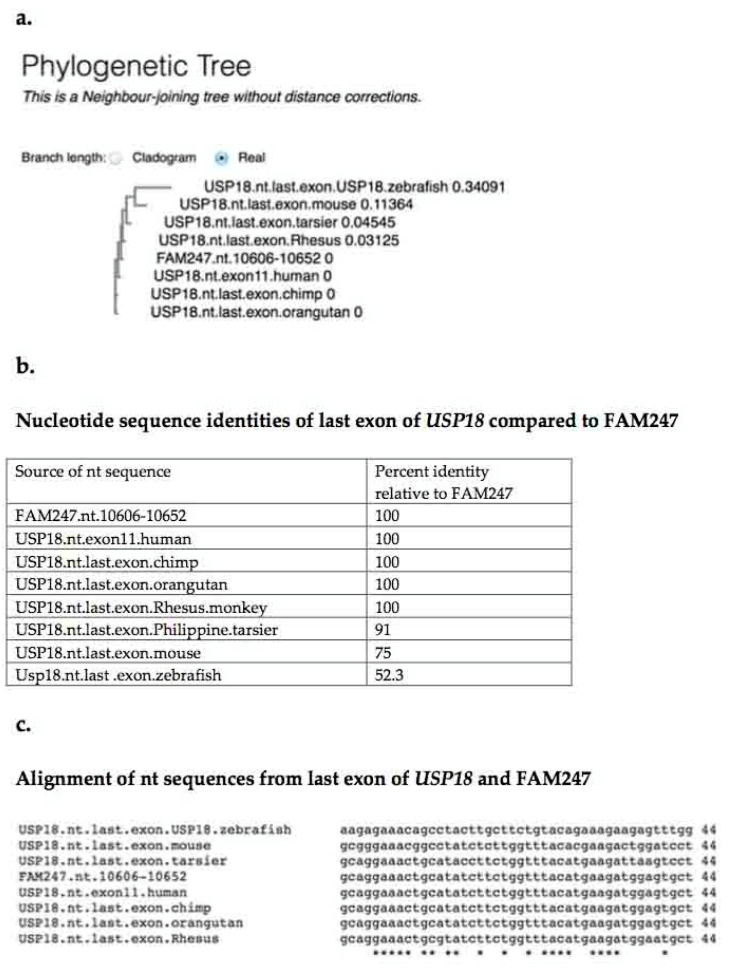
Alignment of the USP18 terminal exon nt sequences from seven species compared with the FAM247 sequence. Data were obtained using the EBI Clustal Omega sequence alignment and phylogeny programs. The EMBL-EBI Clustal Omega Multiple Sequence Alignment program [19] at website http://www.ebi.ac.uk/Tools/msa/clustalo/ was used for nt sequence alignment. (**a**) Phylogenetic tree of USP18 terminal exon sequences from seven species and the FAM247 sequence. (**b**) The percent identities created using Clustal 2.1. (**c**) Alignment of nt sequences. *USP18* gene sequences were accessed from the NCBI website https://www.ncbi.nlm.nih.gov/gene [17].

Both human exon 11, which encodes the last 14 aa (the carboxy terminal end) of the USP18 peptidase, and the 3′UTR of the USP18 mRNA sequence are provided by the FAM247 sequence [8]. The identity between the FAM247 nt sequence and the human/primate exon11 nt sequences is 100%, with the exception of that of *Philippine tarsier* (Figure 4). The sequence of the carboxy terminal exon is more stable than that of the sequence of the entire gene (compare with Table 2). The identities of the *USP18* 3′UTR sequences from various species, compared to FAM247 (Table 3), shows the 3′UTR sequence is also conserved in primates, but to a lesser extent than that of exon 11, and is more similar to the *USP18* gene.

**Figure 5:**
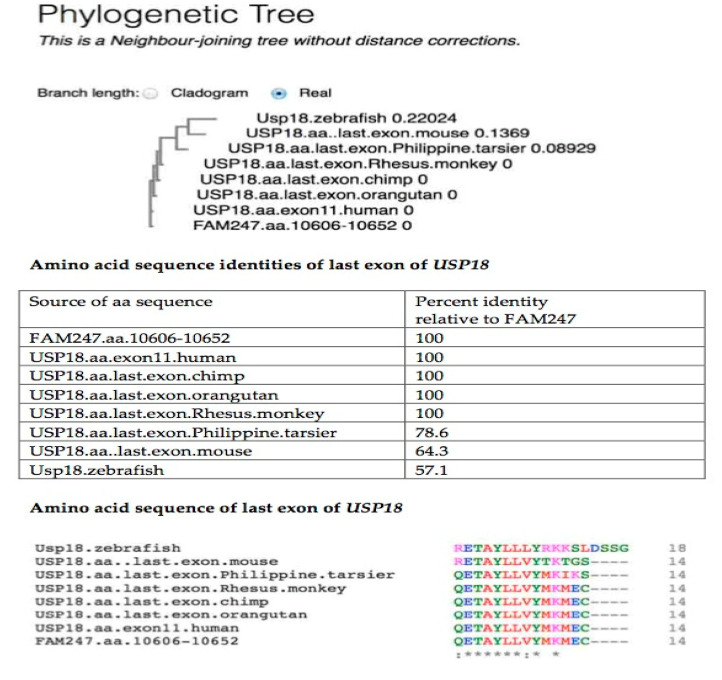
Alignment of the USP18 terminal exon amino acid sequences from seven species compared with the FAM247 translated amino acid sequence. Data were obtained using the EBI Clustal Omega sequence alignment and phylogeny programs. The EMBL-EBI Clustal Omega Multiple Sequence Alignment program [19] at website http://www.ebi.ac.uk/Tools/msa/clustalo/ was used for aa sequence alignment. (**Top**) Phylogenetic tree of USP18 terminal exon aa sequences from seven species and the FAM247 aa sequence. (**Middle**) The percent identities were created using Clustal 2.1. (**Bottom**) Alignment of aa sequences.

Figure 5 shows the USP18 aa sequence percent identity, sequence alignments, and a phylogenetic tree produced from an alignment of *USP18* terminal exon aa sequences from different species with the translated aa sequence of FAM247. Eight of the 14 amino acid residues that form the terminal exon are totally conserved from primates to zebrafish, together with the FAM247 translated aa sequence (Figure 5, bottom). The USP18 carboxy terminal peptide sequence interacts with the INFAR2 interferon receptor, and this sequence is an important regulator of IFN signaling [12]; in addition, the carboxyl end sequence functions in delSGlyation [13,20,21]. A mutation in L365 in the exon 11 sequence _359_QETAYLL_365_VYMKMEC_372_ abolishes deISGylation and INAFR2 binding [20,21]; L_365_ is one of the evolutionarily conserved amino acids of exon 11 (Figure 5). The mutation may alter the protein conformation that is necessary for USP18 to function. On the other hand, the high number of aa residues conserved, relative to the FAM247 translated aa sequence, further supports the proposal that the FAM247 sequence was present in zebrafish *USP18*.

The nt sequence similarity of 52% between FAM247 and zebrafish last exon (Figure 4b), the presence of a number of invariant nt positions (Figure 4c), and the similarity with the 3′UTR sequence (53%; Table 3) suggests that this part of the FAM247 sequence was present in the *USP18* sequence of zebrafish. The invariant nt residues of exon 11, e.g., positions nt 5–9 (Figure 4c), may relate to the functional importance of the *USP18* carboxy terminal end in its role in the regulation of the immune system by *USP18* [12,13]. These invariant nt positions may give a picture, albeit a small picture, of what the ancient FAM247 looked like.

### 4.3. GGT5

**Figure 6:**
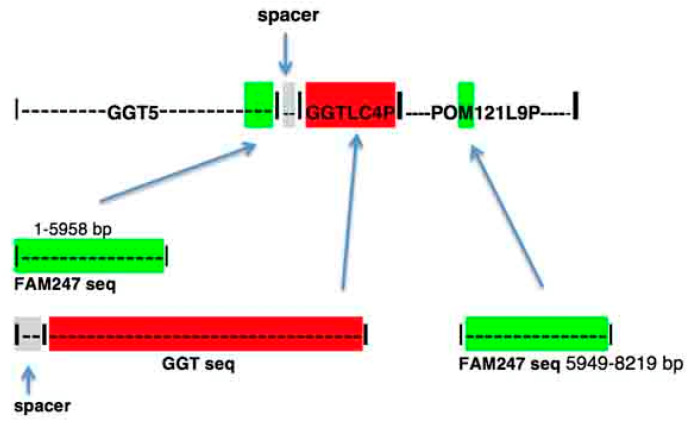
The genes linked to *GGT5* in LCR22G with nearest neighbor arrangements (top schematic) and the source of sequences found in human-linked genes *GGT5–GGTLC4P–POM121L9P*.

The human *GGT5* protein gene resides in chromosomal segmental duplication LCR22G and is linked to pseudogene *POM121L9P* with a spacer sequence, and the pseudogene *GGTLC4P* situated between *GGT5* and *POM121L9P* (Figure 6) [9]. The *GGT5* nearest gene/sequence arrangement is more complex than that of the signatures shown in Figure 1b. *GGT5* is an anomaly as its sequence does not stem from a GGT locus, as other GGT family members do, but from the chromosomal site containing the FAM247 sequence [9]. *GGT5* carries a sequence homologous to the 5′ half of the FAM247A sequence, bp positions 1–5958 (Figure 2 and Figure 6), and *POM121L9P* contains part of the 3′ half of FAM247 (bp 5949–8219). The FAM247 fragments are where there are or were *Alu* sequences. The *GGTLC4P* pseudogene derives its sequence from GGT (Figure 6).

**Figure 7:**
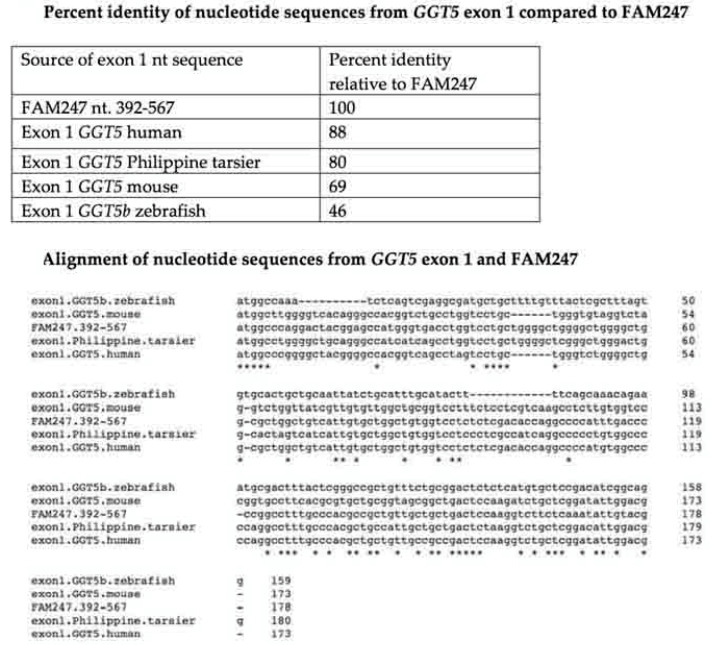
(**Top**) The percent identity of the FAM247 sequence with that of *GGT5* exon 1 sequences from four species. Data were obtained using Clustal 2.1. (**Bottom**) Alignment of the *GGT5* exon nucleotide sequences from the four species, compared with the FAM247 sequence, positions 192–567. Data were obtained using the EBI Clustal Omega sequence alignment and phylogeny programs. The EMBL-EBI Clustal Omega Multiple Sequence Alignment program [19] is at website http://www.ebi.ac.uk/Tools/msa/clustalo/.

*GGT5* and *POM121L9P* appear to have been formed at very different evolutionary times. FAM247 is part of the *GGT5* genes that are in nonhuman primates, including *Philippine tarsier*. In addition, FAM247 provides the sequence found in exon 1 of *GGT5.* There is a significant similarity between the FAM247 nt sequence and that of the mouse *GGT5* exon 1 (Figure 7, top). There is not enough evidence to suggest that the mouse *GGT5* contains the entire 5′ half of the FAM247 sequence, but the alignment of the mouse exon 1 nt sequence with FAM247 shows that a significant number of nucleotides are invariant (Figure 7, bottom). Although there is invariance in 50 out of 173 nt between the FAM247 sequence and zebrafish *GGT5* exon 1, the zebrafish exon sequence shows significant differences, which makes it difficult to further assess a sequence similarity. The exon 1 data are consistent with the formation of the *GGT5* gene with the FAM247 sequence that occurred before the evolutionary appearance of primates and appearing in mice.

### 4.4. Pseudogene POM121L9P

**Figure 8:**
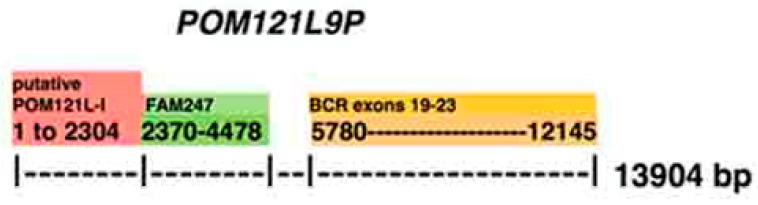
A schematic of the compositional make-up of the pseudogene *POM121L9P*. The numbers under the motifs shown represent the bp positions on the *POM121L9P* sequence. The FAM247 sequence that forms part of *POM121L9P* consists of FAM247 positions 5949–8219, where there are *Alu* sequences at both ends.

*POM121L9P* has a very different sequence compared to the other POM121LP family pseudogenes, and it is a unique sequence. A schematic of the compositional make-up of the *POM121L9P* gene shows that it contains most of the sequence homologous to the putative parent gene, protein gene *POM121L1* (LOC101929738 putative POM121-like protein 1, 2379 bp) on its 5′ side, and the *BCRP1* pseudogene sequence (that is homologous to the 3′ section of the *BCR* gene that includes *BCR* terminal exons 19–23) on its 3′ side (Figure 8). FAM247 may have formed a nucleation site for the addition of these motifs, which are copies of sequences from different regions of the genome, and developed the *POM121L9P* gene. The sequence motifs are found attached to 5′ and 3′ ends of FAM247 at FAM247 bp positions where there are *Alu* sequences (FAM247 bp positions 5949 and 8219). Additionally, the complete *POM121L-1* sequence has an *Alu* sequence at bp positions 2309–2379 (the end of *POM121L-1* is position 2304, at the attachment site with FAM247). *Alu* sequences may have facilitated the addition of *POM121L-1* to FAM247. The BCR sequence addition to *POM121L9P* is more complex as there is an undefined sequence between the two (bp positions 4479–5779 on *POM121L9P*), and there are no *Alu* sequences detected in the BCR sequence at the junction site. The human *POM121L9P* pseudogene RNA transcript is highly expressed in somatic testis tissue, and there is a broad expression of circular RNAs in developing fetal tissues with major expression in lung and adrenal tissues [15,16]. Its functions are not known, but they should be of interest in view of the strong RNA expression levels.

**Figure 9:**
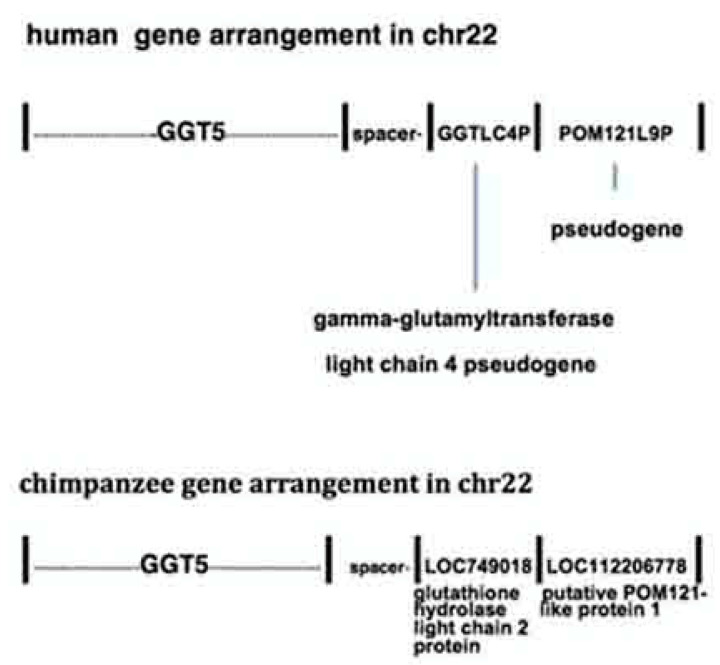
Nearest neighbor gene arrangements in human and chimpanzee chromosomal loci where the *GGT5* gene resides.

In a homologous nearest neighbor gene arrangement that is present in chimpanzee chr22, the genes are annotated as glutathione hydrolase light chain 2 gene (*LOC749018*) and putative POM121-like protein 1 gene (*LOC112206778*); these are linked to *GGT5* through the spacer sequence (Figure 9). Thus, the human pseudogenes *GGTLC4P* and *POM121L9P* sequences are annotated as protein genes in the homologous chromosomal loci of chimpanzee. This is another example of human ncRNA gene sequences that are annotated as protein genes in nonhuman primates, but the isolation of protein products from the chimpanzee genes is essential to add any significance to it. Of importance is that 69% of the *POM121L9P* sequence is present in the chimpanzee genome with 98% identity at the genomic region where there is evolutionary synteny with the comparable chromosomal locus that resides in chimpanzee. There are no FAM247 or POM121L9P sequences that have been found linked to *GGT5* in Rhesus. It appears that the development of the *POM121L9P* sequence may have begun in the chimpanzee but with a partial sequence.

### 4.5. Pseudogenes BCRP3 and POM121L10P

Human pseudogenes *BCRP3* and *POM121L10P* are linked to *GGT1* in the gene/sequence arrangement *GGT1*-spacer-*BCRP3-POM121L10P*, which is present in chr22 LCR22H. FAM247 forms part of the two pseudogenes: *BCRP3*, which has the FAM247 positions 33–5958 and *POM121L10*, positions 5957–8219 (Figure 2). Thus, parts of the 5′ and 3′ regions of FAM247 are found in these linked genes, which is similar to the presence of FAM247 in genes *GGT5* and *POM121L9P*.

**Figure 10:**
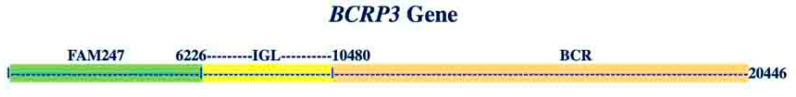
Sequence motif of the *BCRP3* gene. Positions 6226–10480 of *BCRP3* span the IGL insert. The total length of *BCRP3* is 20446 bp.

*BCRP3* is a member of the *BCRP* pseudogene family consisting of eight pseudogenes, all of which contain the homologous sequence of the 3′ end sequence of the *BCR* protein gene except for *BCRP8*. *BCRP3* is one of the family members that differs as it contains additional sequence motifs (Figure 10) and is the only *BCRP* family member that contains the FAM247 sequence. The *BCRP3* gene appears to have a unique sequence. The compositional make-up of *BCRP3* shows that its 5′ side has the FAM247 sequence, which is followed by a 4255-bp segment of the immunoglobulin lambda locus (IGL) and the 3′ end of the BCR sequence (Figure 10). The IGL sequence (nt positions 590,381–594,292) from the Homo sapiens immunoglobulin lambda locus (IGL) on chromosome 22) [17] is homologous to the IGL locus V segments and three C segments, which are not known to encode immunoglobulin proteins. The IGL sequence has an *Alu* sequence at the junction with FAM247, which may relate to the process of attachment of IGL to FAM247 during the maturation of the pseudogene. The IGL sequence is made up entirely of 8 *Alu* sequences, 7 LINE elements, other transposable elements, and small repeats (http://www.repeatmasker.org/cgi-bin/WEBRepeatMasker). In terms of RNA expression, the pseudogene shows a broad expression of linear RNA in 27 normal somatic tissues and a broad expression of circular RNA in developing fetal tissues [15,16].

The *POM121L10P* sequence is linked to *BCRP3* on chr22. It also contains the FAM247 sequence (Figure 2). *POM121L10P* is compositionally made up of nearly the entire sequence of the related pseudogene *POM121L1P* but has a 1062-bp sequence at its 3′ end that consists of a copy of the 3′ end of the BCR gene. *POM121L10P* also appears to be a unique gene construct. The *POM121L10P* linear RNA transcript is strongly expressed in testes; circular RNAs are broadly expressed in fetal tissues. [15,16]. Thus, both this gene and *BCRP3* show a robust RNA expression. It should be pointed out that there are additional *POM121LP* pseudogene family members that carry the FAM247 sequence, but they are not addressed here.

In the Rhesus genome, some genes/neighbor sequences display synteny. The Rhesus *GGT1* is linked to the spacer sequence and followed by the FAM247 sequence, which is similar to that of the human *GGT1* gene/sequence arrangement. Rhesus gene *LOC107000612,* annotated as a “breakpoint cluster region protein-like” is situated close to *GGT1*. This is part of the homologous chromosomal region where the pseudogene *BCRP3* resides in the human genome; approximately 78% of the human *BCRP3* sequence is present in the Rhesus genome at this locus. The *BCRP3* sequence has not been detected in the early primate *Philippine tarsier*. Thus, the earliest appearance of the *BCPR3* sequence is in the Rhesus species, and the sequence appeared to have matured into a pseudogene in humans. There was a large chromosomal expansion of the Rhesus monkey genome between genes *GGT1* and *GGT5*. The chromosomal length between genes *GGT1* and *GGT5* in *Philippine tarsier* is 2872 bp; in the rhesus monkey, it is 216,200 bp. Thus, there is a 75-fold sequence expansion between *GGT1* and *GGT5* in Rhesus. Segments of the *BCRP3* gene may have formed with this chromosomal expansion. This may account for the source of the *BCRP3* sequence in Rhesus, but again, the sequence is not found in *Philippine tarsier*.

Using Figure 8 and Figure 10, a model of de novo gene development from protogene sequences can be visualized whereby the FAM247 sequence, which is present in genomic regions that display synteny, forms nucleation sites where other genomic sequences are added during the maturation process to complete the pseudogene structure.

## 5. Conclusions

Both the FAM247 lincRNA gene family and pseudogenes appear to have the FAM247 sequence as a foundation for gene development; however, the mechanism of formation and the compositional make-up between lincRNA genes and pseudogenes greatly differ. The *FAM247* family (as well as the *FAM230* lincRNA gene family) was formed by gene duplication and family members display sequences that are “variations on a theme”. Although pseudogenes *BCRP3*, *POM121L9P*, and *POM121L10P* contain duplications of part of or entire portions of parent protein genes, they were formed differently by a de novo process of addition of large unrelated genomic sequences to the FAM247 sequence, with the resultant formation of unique pseudogene sequences. *Alu* elements are present in FAM247 at sites of attachment, and these may contribute to the process of sequence addition, possibly by *Alu/Alu* recombination. As these pseudogenes are unique, with large sequences unrelated to the parent protein genes, the question is whether they should be called pseudogenes. How *USP18* and *GGT5* protein genes developed is not known, but a putative ancient FAM247 sequence was likely involved. A separate but important aspect of the FAM247 sequence in cellular and molecular functions is that it contributes the amino acid sequence for protein exons, the first exon of *GGT5* and the last exon of *USP18*. The functions of the carboxy terminal aa sequence of *USP18* are of major significance because of the important role in the regulation of the immune system.

A search for possible nucleation elements similar to FAM247 is important in order to determine the prevalence of this type of protogene. An analysis of repeat sequences that are part of phylogenetically conserved nearest-neighbor genes/sequences in human chromosomes that have a large number of segmental duplications, e.g., chr 15, 16, and X [22], may help find other gene forming elements. Blat searches with the Ensembl program using lncRNA gene sequences as a query can help locate sequences shared with other genes, including protein genes. Use of the NCBI Blast/align two sequences program may reveal small sequence segments that are present in diverse genes.

## Figures and Tables

**Table 1:** Human genes and gene families found in linked sequences: FAM230–FAM247–GGT, FAM230–USP18.

Gene/Gene Family	Type	Locus Origin
* *FAM230A-J*	lincRNA	FAM230
*FAM247A-D*	lincRNA	FAM247
*POM121L9P*, *POM121L10P*	pseudogene	FAM247
*BCRP3*	pesudogene	FAM247
*GGT1*, *GGT2*	protein	GGT
*GGTLC2*	protein	GGT
*GGTLC3*	protein	GGT
*GGT3P*	pseudogene	GGT
*GGT4P*	pseudogene	GGT
*GGTLC4P*	pseudogene	GGT
*GGTLC5P*	pseudogene	GGT
*GGT5*	protein	FAM247
*USP18*	protein	FAM247

* Some FAM230 family members, such as FAM230J, do not have the linked sequence signatures.

**Table 2:** *USP18* gene and protein sequence identities and evolutionary time between species.

Species	*USP18* Gene nt Sequence %Identity	USP18 aa Sequence %Identity	Evolutionary Age (MYA) *
human	100%	100%	0 MYA
chimpanzee	99%	99%	6 MYA
Rhesus monkey	92%	94%	25 MYA
*Philippine tarsier*	66%	80%	50 MYA
mouse	51%	71%	90 MYA
zebrafish	39%	31%	350 MYA

* approximate age in million years ago (MYA) [18].

**Table 3:** Nucleotide sequence identities of 3′UTR of *USP18* from different species.

Source of nt Sequence	% Identity Relative to FAM247 3′ End
FAM247A 3′ end nt 10,653–11,231	100
USP18 3′UTR human	99.8
USP18 3′UTR chimp	98.6
USP18 3′UTR Rhesus monkey	90.2
USP18 3′UTR *Philippine tarsier*	71.9
USP18 3′UTR mouse	49.5
USP18 3′UTR zebrafish	53.1

## References

[1] Ohno S. (1999). Gene duplication and the uniqueness of vertebrate genomes circa 1970–1999. Semin. Cell Dev. Biol..

[2] Jacob F. (1977). Evolution and tinkering. Science.

[3] Wang W., Yu H., Long M. (2004). Duplication-degeneration as a mechanism of gene fission and the origin of new genes in Drosophila species. Nat. Genet..

[4] Carvunis A.R., Rolland T., Wapinski I., Calderwood M.A., Yildirim M.A., Simonis N., Charloteaux B., Hidalgo C.A., Barbette J., Santhanam B. (2012). Proto-genes and de novo gene birth. Nature.

[5] McLysaght A., Guerzoni D. (2015). New genes from non-coding sequence: The role of de novo protein-coding genes in eukaryotic evolutionary innovation. Philos. Trans. R. Soc. B Biol. Sci..

[6] Schlotterer C. (2015). Genes from scratch—The evolutionary fate of de novo genes. Trends Genet..

[7] Van Oss S.B., Carvunis A.R. (2019). De novo gene birth. PLoS Genet..

[8] Ulitsky I., Bartel D.P. (2013). lincRNAs: Genomics, evolution, and mechanisms. Cell.

[9] Delihas N. (2020). Formation of human long intergenic non-coding RNA genes, pseudogenes, and protein genes: Ancestral sequences are key players. PLoS ONE.

[10] Nowell P., Hungerford D. (1960). A minute chromosome in human chronic granulocytic leukemia. Science.

[11] De Klein A., van Kessel A.G., Grosveld G., Bartram C.R., Hagemeijer A., Bootsma D., Spurr N.K., Heisterkamp N., Groffen J., Stephenson J.R. (1982). A cellular oncogene is translocated to the Philadelphia chromosome in chronic myelocytic leukaemia. Nature.

[12] Arimoto K.I., Löchte S., Stoner S.A., Burkart C., Zhang Y., Miyauchi S., Wilmes S., Fan J.B., Heinisch J.J., Li Z. (2017). STAT2 is an essential adaptor in *USP18*-mediated suppression of type I interferon signaling. Nat. Struct. Mol. Biol..

[13] Honke N., Shaabani N., Zhang D.E., Hardt C., Lang K.S. (2016). Multiple functions of *USP18*. Cell Death Dis..

[14] Bruford E.A., Braschi B., Denny P., Jones T.E.M., Seal R.L., Tweedie S. (2020). Guidelines for human gene nomenclature. Nat. Genet..

[15] Szabo L., Morey R., Palpant N.J., Wang P.L., Afari N., Jiang C., Parast M.M., Murry C., Laurent L.C., Salzman J. (2015). Statistically based splicing detection reveals neural enrichment and tissue-specific induction of circular RNA during human fetal development. Genome Biol..

[16] Fagerberg L., Hallström B.M., Oksvold P., Kampf C., Djureinovic D., Odeberg J., Habuka M., Tahmasebpoor S., Danielsson A., Edlund K. (2014). Analysis of the human tissue-specific expression by genome-wide integration of transcriptomics and antibody based proteomics. Mol. Cell. Proteom..

[17] O’Leary N.A., Wright M.W., Brister J.R., Ciufo S., Haddad D., McVeigh R., Rajput B., Robbertse B., Smith-White B., Ako-Adjei D. (2016). Reference sequence (RefSeq) database at NCBI: Current status, taxonomic expansion, and functional annotation. Nucleic Acids Res..

[18] Siepel A. (2009). Phylogenomics of primates and their ancestral populations. Genome Res..

[19] Madeira F., Park Y.M., Lee J., Buso N., Gur T., Madhusoodanan N., Basutkar P., Tivey A.R.N., Potter S.C., Finn R.D. (2019). The EMBL-EBI Search and Sequence Analysis Tools APIs in 2019. Nucleic Acids Res..

[20] Malakhov M.P., Malakhova O.A., Kim K.I., Ritchie K.J., Zhang D.E. (2002). Protein ISGylation Modulates the JAK-STAT Signaling Pathway. J. Biol. Chem..

[21] Dauphinee S.M., Richer E., Eva M.M., McIntosh F., Paquet M., Dangoor D., Burkart C., Zhang D.E., Gruenheid S., Gros P. (2014). Contribution of increased ISG15, ISGylation and deregulated type I IFN signaling in Usp18 mutant mice during the course of bacterial infections. Genes Immun..

[22] Redaelli S., Maitz S., Crosti F., Sala E., Villa N., Spaccini L., Selicorni A., Rigoldi M., Conconi D., Dalprà L. (2019). Refining the phenotype of recurrent rearrangements of chromosome 16. Int. J. Mol. Sci..

